# Comparison of Spinal Cord Stimulation vs. Dorsal Root Ganglion Stimulation vs. Association of Both in Patients with Refractory Chronic Back and/or Lower Limb Neuropathic Pain: An International, Prospective, Randomized, Double-Blinded, Crossover Trial (BOOST-DRG Study)

**DOI:** 10.3390/medicina58010007

**Published:** 2021-12-21

**Authors:** Philippe Rigoard, Manuel Roulaud, Lisa Goudman, Nihel Adjali, Amine Ounajim, Jimmy Voirin, Christophe Perruchoud, Bénédicte Bouche, Philippe Page, Rémy Guillevin, Mathieu Naudin, Martin Simoneau, Bertille Lorgeoux, Sandrine Baron, Kevin Nivole, Mathilde Many, Iona Maitre, Raphaël Rigoard, Romain David, Maarten Moens, Maxime Billot

**Affiliations:** 1 PRISMATICS Lab (Predictive Research in Spine/Neuromodulation Management and Thoracic Innovation/Cardiac Surgery), Poitiers University Hospital, 86021 Poitiers, France; Manuel.ROULAUD@chu-poitiers.fr (M.R.); Nihel.adjali@chu-poitiers.fr (N.A.); Amine.OUNAJIM@chu-poitiers.fr (A.O.); dr.bouche@gmail.com (B.B.); Bertille.LORGEOUX@chu-poitiers.fr (B.L.); sandrine.baron@chu-poitiers.fr (S.B.); Kevin.NIVOLE@chu-poitiers.fr (K.N.); mathilde.many@chu-poitiers.fr (M.M.); iona.maitre@etu.univ-poitiers.fr (I.M.); romain-david@hotmail.fr (R.D.); Maxime.BILLOT@chu-poitiers.fr (M.B.); 2 Department of Spine Surgery & Neuromodulation, Poitiers University Hospital, 86021 Poitiers, France; Philippe.PAGE@chu-poitiers.fr; 3 Pprime Institute UPR 3346, CNRS, ISAE-ENSMA, University of Poitiers, 86360 Chasseneuil-du-Poitou, France; 4 Department of Neurosurgery, Universitair Ziekenhuis Brussel, 1090 Brussels, Belgium; lisa.goudman@gmail.com (L.G.); mtmoens@gmail.com (M.M.); 5 STUMULUS Research Group, Vrije Universiteit Brussel, 1090 Brussels, Belgium; 6 Department of Neurosurgery, Hopitaux Civils de Colmar, 68000 Colmar, France; jimmy.voirin@ch-colmar.fr; 7 Service of Anesthesiology and Pain Centre, University Hospital of Lausanne (CHUV), 1011 Lausanne, Switzerland; christophe.perruchoud@chuv.ch; 8 Department of Radiology, Poitiers University Hospital, 86021 Poitiers, France; remy.guillevin@chu-poitiers.fr (R.G.); mathieu.naudin@chu-poitiers.fr (M.N.); 9 UMR CNRS 7348, DACTIM-MIS/LMA Laboratory, University of Poitiers, 86000 Poitiers, France; 10 Department of Kinesiology, Faculty of Medicine, Laval University, Quebec, QC G1V 0A6, Canada; martin.simoneau@kin.ulaval.ca; 11 Centre Interdisciplinaire de Recherche en Réadaptation et Intégration Sociale (CIRRIS), Quebec, QC G1M 2S8, Canada; 12 CEA Cadarache, Département de Support Technique et Gestion, Service des Technologies de l’Information et de la Communication, 13108 Saint-Paul-Lez-Durance, France; cydran@gmail.com; 13 Department of Physical and Rehabilitation Medicine, Poitiers University Hospital, University of Poitiers, 86021 Poitiers, France

**Keywords:** neurostimulation, burst, leg pain, refractory pain, failed back surgery syndrome, hybrid stimulation, energy consumption

## Abstract

While spinal cord stimulation (SCS) is a well-established therapy to address refractory persistent spinal pain syndrome after spinal surgery (PSPS-T2), its lack of spatial selectivity and reported discomfort due to positional effects can be considered as significant limitations. As alternatives, new waveforms, such as burst stimulation and different spatial neural targets, such as dorsal root ganglion stimulation (DRGS), have shown promising results. Comparisons between DRGS and standard SCS, or their combination, have never been studied on the same patients. “BOOST DRG” is the first prospective, randomized, double-blinded, crossover study to compare SCS vs. DRGS vs. SCS+DRGS. Sixty-six PSPS-T2 patients will be recruited internationally in three centers. Before crossing over, patients will receive each stimulation modality for 1 month, using tonic conventional stimulation. After 3 months, stimulation will consist in switching to burst for 1 month, and patients will choose which modality/waveform they receive and will then be reassessed at 6 and 12 months. In addition to our primary outcome based on pain rating, this study is designed to assess quality of life, functional disability, psychological distress, pain surface coverage, global impression of change, medication quantification, adverse events, brain functional imaging and electroencephalography, with the objective being to provide a multidimensional insight based on composite pain assessment.

## 1. Introduction

Spinal cord stimulation (SCS) is a well-established therapy to alleviate severe intractable neuropathic pain [1,2,3,4,5,6,7] by improving pain control and quality of life [8,9] in patients with failed back surgery syndrome (FBSS) or persistent spinal pain syndrome type 2 (PSPS-T2) [10]. Using this reversible treatment option, the prerequisite to address pain with conventional therapy modality is to obtain appropriate coverage of the painful area, by inducing paresthesia on corresponding painful territories [1,8,11]. Despite its effectiveness, SCS has some limitations.

First, pauci-discriminative stimulation of billions of sensory epicritic fibers of the dorsal columns using electrical impulses makes it very complex for SCS to be selective in terms of pain coverage for patients [11]. This lack of selectivity, associated with the type of implanted lead, its positioning, its properties, patient variability and the challenge to provide adequate spatial and temporal neural targeting using electrical source-specific capabilities, can lead, on the one hand, to feel paresthesia in a non-painful area and, on the other hand, to not be able to cover decisive painful body regions and/or pain trigger(s), particularly in focal distal pain distribution, as in foot and groin pain [12]. As described by Holsheimer et al. [13], lead positioning can dramatically impact paresthesia coverage and energy consumption. New available paresthesia-free waveforms, such as burst, high frequency or high dose, appear to limit this problem by suppressing patient perception of the therapy [14,15,16,17,18,19]. However, they might also result in significantly higher energy consumption, requiring more frequent recharges, or lead to premature battery replacement. Third, a decrease/loss of effectiveness may occur over time [8] due to neural plasticity. This might explain why, up until now, physicians have been obsessively focusing on pain coverage, expending considerable energy to blur nociceptive signals by stimulating long sensory fibers with various SCS spatial programming strategies [4], rather than attempting to stimulate other potential pain generators. One major target appears to be located at the dorsal root ganglion (DRG) level and plays a crucial role in pain generation [20,21]. To validate this hypothesis, medical device and neuromodulation industries have developed DRG stimulation (DRGS) [22]. DRGS appears to be a promising technology for patients suffering from refractory chronic neuropathic pain, and particularly focal pain, since it operates more selectively on the nervous system [22,23,24,25,26,27,28]. Furthermore, DRGS could have an advantage over SCS by processing less positional sensitivity, possessing more stability and superior electrical efficiency in terms of energy consumption. Due to its proximity and lack of cerebro-spinal fluid (CSF), it requires far less amplitude to deliver therapy to the appropriate neural target [13,29]. In a recent high-quality prospective, multicenter, randomized comparative trial conducted on 152 subjects implanted with either an SCS or a DRG stimulation system, Deer et al. [24] reported that DRGS leads to a significantly higher rate of treatment success (≥50% pain relief) compared with conventional SCS (81.2% vs. 56.7%, respectively) in patients with complex regional pain syndrome (CRPS) and/or causalgia. The authors also reported that, using DRGS, pain relief was maintained through 12 months of follow-up and was greater than with SCS. However, in this study, SCS and DRGS were assessed in two distinct groups of CRPS patients, between whom comparison was not possible. Some might not have responded to SCS or DRGS due to their baseline latent confounding variables rather than therapy efficacy variability. Furthermore, synergy between SCS and DRGS working simultaneously on the same patient might catalyze therapy outcome by optimizing spatial and temporal neural tissue targeting, pain coverage (i.e., selectivity), pain relief, cortical perception and, ultimately, electrical consumption. This has never been documented so far.

In parallel with neural spatial targeting improvements, new available waveforms including burst paradigms have changed the modulation of the temporal resolution of the signal, allowing for “paresthesia-free” stimulation [30,31]. Recent clinical trials have provided evidence of burst-SCS efficacy in PSPS-T2 patients [32,33,34,35]. However, to date, only animal studies have demonstrated the effectiveness of burst applied to DRG stimulation [36,37]. The opportunity to apply burst waveform to DRG could help to refine the design of the next generation of internal pulse generators (IPGs) and to explore new mechanisms of action. To our knowledge, DRGS combined with burst has yet to be reported in humans.

In a recent review, Linderoth and Foreman [38] presented the current state of knowledge regarding conventional SCS mechanisms of action, by indicating that gamma-aminobutyric acid (GABA) release and GABA-B receptors are pivotal in suppressing glutamate release and consequently relieve pain [39,40,41,42]. Other animal studies have reported that cholinergic activation can enhance SCS pain relief [43,44]. Similarly, release of neurotransmitters such as adenosine, serotonin and noradrenaline by SCS could contribute to pain relief [43,45,46]. Recent studies have reported that 50% of the effects of conventional SCS in neuropathic pain imply supraspinal circuitry [45,47]. Assuming spinal and supraspinal activation during SCS or DRGS applied in an animal model [47], a recent systematic review synthesizes current literature on the supraspinal mechanisms of action underlying the pain-relieving effect of SCS and DRGS in animal and human studies [46]. Through 54 studies, Goudman et al. [46] reported that three main supraspinal hypotheses indicate modulation of the descending nociceptive inhibitory pathways, followed by modulation of the ascending medial and lateral pathways. The difference, in terms of mechanisms of action, between tonic conventional stimulation (TCS) and burst stimulation could be due to the more selective modulation of the medial pain pathway provided by burst stimulation, as evidenced on metabolic activation of the dorsal anterior cingulated cortex on a limited number of patients [48]. Moreover, TCS significantly increases the spontaneous activity of neurons in the gracile nucleus, which are known to project to the primary somatosensory cortex in the brain. Increased spontaneous activation of the spinal nucleus might account for the sensation of paresthesia that typically occurs during TCS, but not burst stimulation [49]. Combining electroencephalography (EEG) and functional magnetic resonance imaging (fMRI) to test these hypotheses, we gain insight on supraspinal mechanisms of action of SCS and DRGS, including not only tonic conventional stimulation (TCS), but also burst stimulation modalities.

To address these challenges, we designed a randomized controlled trial (RCT) with a 3-month crossover design, where SCS, DRGS or SCS+DRGS (DUAL) will be proposed to a single patient presenting with refractory chronic lower limb neuropathic pain (e.g., CRPS, diabetic foot peripheral neuropathy, foot and ankle peripheral neuropathy, radicular pain associated with PSPS-T2) and/or documented neuropathic refractory back pain. After the crossover period, all patients will be switched to burst waveform with 1-month follow-up, and at 4-month follow-up, they will be asked to choose SCS, DRGS or SCS+DRGS with tonic conventional or burst waveform.

### Aims and Objectives

The BOOST DRG study is a randomized, controlled trial comparing the efficacy of SCS vs. DRGS vs. combination of SCS+DRGS (DUAL) and TCS vs. burst in a crossover design in patients with chronic lower limb neuropathic pain and/or neuropathic back pain.

The primary objective of this study is to compare the efficacy of SCS vs. DRGS vs. DUAL on global pain relief in patients with chronic lower limb neuropathic pain and/or neuropathic back pain.

Secondary objectives are to compare pain intensity, pain surface and paresthesia coverage surface to assess positional or postural effects on paresthesia, to compare changes in quality of life, functional disability and psychological state, to assess patient satisfaction and, finally, to compare the effects of stimulation modalities on brain imaging based on fMRI and EEG. Using this study data, we will extract baseline factors associated with long-term stimulation efficacy. These predictors will be included in a predictive model.

## 2. Materials and Methods

This protocol is reported in accordance with the SPIRIT 2013 guidelines for protocols of clinical trials [50].

### 2.1. Design and Setting

The BOOST DRG study is divided into two phases.

Phase A (Pilot study) is a randomized, double-blinded, crossover, six-arm study evaluating the feasibility and comparing the efficacy of three stimulation modalities: SCS, DRGS and DUAL (SCS+DRGS) on 12 patients during 3-month follow-up, which will be conducted only in Poitiers University Hospital (France). According to the phase A results, in terms of feasibility and safety, a second phase is planned, following the same design and including a larger sample of patients, internationally, to take into account the variability of practices.

Phase B (Main study) is a prospective, multicentric, randomized, double-blinded, crossover, six-arm study comparing the efficacy of three stimulation modalities: SCS, DRGS and DUAL stimulation (SCS+DRGS) on 54 patients, conducted in the Poitiers University Hospital (France), the Colmar Louis Pasteur Hospital (France) and in Genève Tower Hospital Pain Clinic/Lausanne University Hospital (Switzerland).

Phase A and phase B have the same design. Patients will be implanted with the Octrode^TM^ contact percutaneous lead for SCS and one or several Quattrode^TM^ lead(s) for the DRGS, enabling use of the three different stimulation modalities in the same patient. The leads will be connected to a single external pulse generator. Patients will be randomized in 6 different arms in a 1:1:1:1:1:1 ratio: SCS alone, DRGS alone and DUAL stimulation. During the 3-month crossover period, patients will be under the tonic conventional stimulation (TCS) waveform, whatever the stimulation modality. Each stimulation modality will be administered for 1 month. Patients will be assessed at the end of each stimulation modality period, corresponding to the 1-month, 2-month and 3-month follow-up visits. At the end of the crossover period, stimulation modality will not be changed and will be switched to the burst waveform for 1 month. Patients will be assessed at the end of this 1-month period, corresponding to the study’s 4-month follow-up visit. Patients will then decide which stimulation modality and waveform they prefer and will be assessed at 6 months and 12 months.

**Figure 1:**
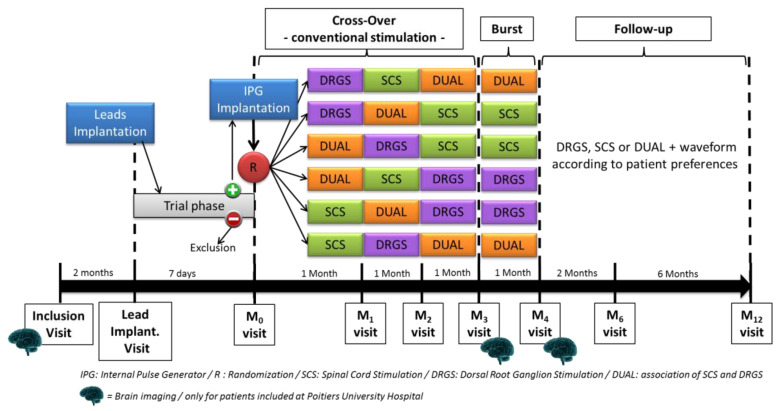
BOOST DRG study design (applicable for phase A and phase B).

Patient flow and the study design are presented in Table 1 and Figure 1, respectively.

### 2.2. Patient Selection

The study population comprises patients suffering from chronic lower limb neuropathic pain and/or neuropathic back pain. A patient must meet all inclusion criteria and none of the non-inclusion criteria and the exclusion criteria to be eligible for the study.

#### 2.2.1. Inclusion Criteria

The patient, aged between 18 and 80 years old, presents with refractory chronic lower limbs neuropathic pain or/and neuropathic back pain for at least 6 months, with VAS ≥ 5/10. The patient’s symptoms are stable for at least 30 days. Pain medication dosage(s) are stable for at least 30 days and the patient is refractory to other treatment modalities (e.g., medication, psychological therapies, pain interventions, surgery). The patient is eligible for SCS after a pre-implantation assessment by a multidisciplinary team, as described by the French National Authority for Health (Haute Autorité de Santé (HAS)). The patient is covered by French national health insurance. The patient understands and accepts the constraints of the study and has given written consent to the study after receiving clear and complete information.

#### 2.2.2. Non-Inclusion Criteria

The patient has a coagulation disorder, had corticosteroid therapy within the past 30 days, had radiofrequency therapy within the past 3 months, was diagnosed with cancer in the past 2 years or had surgery within the past 6 months. The patient is or has been treated with SCS, subcutaneous or peripheral nerve stimulation, an intrathecal drug delivery system. The patient requires closer protection, i.e., minors, pregnant women, nursing mothers, subjects deprived of their freedom by a court or administrative decision, subjects admitted to a health or social welfare establishment, major subjects under legal protection and, finally, patients in an emergency setting. The patient participates in any interventional study on health product or any study able to interfere with the current study endpoints. Women of childbearing potential not using an effective contraception are not expected to be included in this study. For Poitiers University Hospital only, patients who have magnetic brain imaging (MRI) contraindications will not be included.

#### 2.2.3. Exclusion Criteria

The patient is unable to appropriately use stimulation equipment, the patient has failure of the trial phase, defined as pain reduction <50% between the inclusion visit and 7 days after lead implantation or the patient presents with any medical condition judged as relevant/significant by the investigator.

### 2.3. Interventions

**Figure 2:**
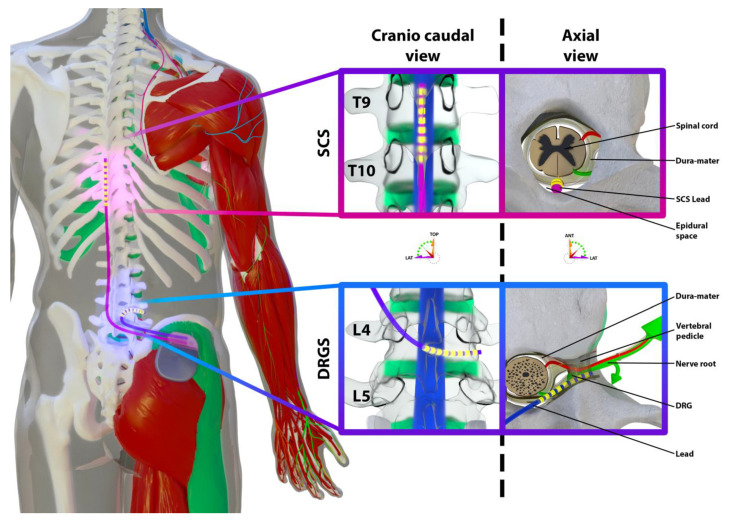
An artistic view illustrating SCS and DRGS implantation.

Subjects who meet all the inclusion criteria and none of the non-inclusion or exclusion criteria will be implanted with the Octrode^TM^ contact percutaneous lead for SCS and one or several Quattrode^TM^ lead(s) for the DRGS, depending on the pain (bi)lateralization and the pain topography (Figure 2).

The following implantation procedure describes the various steps of the surgery until final implantation of the lead. The procedure length is about 90 min.

#### 2.3.1. Phase 1: Lead Implantation

The lead positioning under awake anesthesia will allow for patients to be tested intra-operatively in order to determine the anatomical sweet spot and the optimal paresthesia coverage, according to patient-specific pain distribution. Preliminary electrophysiological testing will be performed on leads and percutaneous leads will then be navigated longitudinally through the following.

The epidural space, to determine the best vertebral level and implantation site, addressing the “sweet spot” location (an anatomical area located within the dorsal column (DC) of the spinal cord, able to provide significant paresthesia generation in the adequate dermatome, if electrically stimulated) [51], during an initial phase of intra-operative testing of the percutaneous SCS lead.

The foramen, using a trans-grade approach [52], to target the DRG(s) and determine the best location of implantation in terms of vertebral level, foramen level and final lead positioning.

Epidural fat and scar tissue formation could play a substantial role in the energy delivered from the IPG to dorsal column or DRG. This will be explored by following impedance throughout the study and will be taken into account as a potential limitation. To reflect daily practice, impedance will be measured at each contact level according to the user’s manual (between 200 and 3000 Ω) in this clinical study. In case of impedance higher than 3000 Ω, the contact will be automatically deactivated. In this case, we will activate contacts with whom the patient will experience the best pain relief. Some TCS patterns will be tested thanks to the patient’s cooperation, by using various predetermined combinations to achieve good pain coverage and validate the “sweet-spot” location. This will be facilitated by an integrated operating room including several of the new computer-assisted and imaging-assisted technologies (multiple splitable screens with wall live projection, full HD camera filming the patient and C-arm monitor, HF microphones and earphones enhancing surgeon/patient per-operative crosstalk, etc.) designing an operative theater [53].

After completion of lead programming testing, the leads will be permanently implanted and secured with appropriate anchoring, at the final vertebral level. Very specific attention will be given to the DRG(s) anchorage. The rest of the procedure will be performed as already described in our previous published works [31,54].

Potential lead migration should be documented by imaging in case the patient experiences inadequate pain relief and/or for coverage after initial positive outcome. In case of lead displacement, documented by X-ray, a revision surgery will be recommended to check hardware dysfunction, including lead migration or fracture. This complication will be reported as an adverse event. Pain rating and outcomes will be analyzed taking the adverse event into account.

#### 2.3.2. Phase 2: SCS+DRGS Trial Phase

During the trial phase, both SCS and DRGS will be used separately and/or simultaneously. The trial will be performed for a period of 7 days to assess the benefits of stimulation according to HAS guidelines prior to definitive implantation of the IPG. IPG implantation will be based on the following criterion: at least 50% reduction of pain assessed with a visual analogic scale (VAS).

Following surgical implantation of the leads, after 24 h of strict bed rest to avoid secondary early lead displacement, lead programming will be performed according to the TCS by testing a series of programming combinations designed to obtain coverage of the painful territory.

In case of SCS/DRGS trial failure, the leads will be removed, and the patient will be managed according to standard practice (including another assessment by the pain physician to define a nonsurgical solution). If the patient is explanted, he/she will be excluded from the study.

#### 2.3.3. Phase 3: IPG Implantation

Subjects who succeed in the lead trial (at least 50% reduction of pain) will receive a PROCLAIM^TM^ System implant, depending on their electrical consumption (per following French ministry of health recommendations and implanting guidelines), and in view of preserving MRI compatibility, so as to be able to explore implanted patients with functional imaging during phase B.

In case of inadequate pain relief, impedance and lead position by radiographic imaging will be performed during the visits planned in the protocol or in supplementary visit. After ensuring that reprogramming cannot achieve pain relief and lead position by radiographic imaging, surgery could be performed to explore the failure to relieve pain by SCS or DRGS such as lead connection, defective lead or scar tissue formation.

To achieve SCS placement, the implanter should place the lead according to the cranio-caudal projection of the conus terminalis to anticipate/estimate a priori optimal lead placement.

### 2.4. Spinal Cord Stimulation Programming Modalities

Different waveforms will be used for the purpose of this study, including TCS and BurstDR^TM^. They differ in terms of amplitude, pulse width and frequencies. TCS delivers a constant stream of pulses:

- Pulse width: between 100 μs and 500 μs;
- Frequencies: between 30 Hz and 100 Hz;
- Amplitude: adapted to individual participant perception, the objective being to achieve a comfortable level of paresthesia. The minimum amplitude step size is 0.1 mA.

BurstDR^TM^ stimulation:

- 40 Hz BurstDR^TM^ mode of constant-current stimuli with 5 spikes at 500 Hz per burst;
- Pulse width and interspike intervals of 1 ms;
- The minimum amplitude step size is 0.05 mA;
- Intermittent dosing: ratio from 1:3 (30-s ON and 90-s OFF) to 1:12 (30 s ON and 360 s OFF) [55].

The stimulation delivered by the IPG to the spinal or dorsal root ganglion target could depend on many parameters. First, it is well documented that CSF thickness varies according to the vertebral level and that the perception threshold is also impacted by the postural changes [56]. Positional effects have been attributed to a combination of factors including the distance between electrodes and the dorsal columns as well as the overall thickness of the CSF layer at the implanted spinal level [29]. These parameters clearly influence the current intensity delivered to the spinal cord [57] and consequently the paresthesia threshold generated by SCS [58]. On the other hand, DRGS is less likely to be impacted by these parameters due to a very thin layer of CSF surrounding the DRG. The distance between the lead and the neural target is quite small and presumably less sensitive to postural changes.

### 2.5. Clinical Assessments

Randomization is carried out at the IPG implantation visit. Subjects are assessed prior to randomization, during the inclusion visit and the lead implantation visit, and at 1-month, 2-month, 3-month, 4-month, 6-month and 12-month follow-up visits. Assessments are performed by appropriately trained and delegated study staff independently from industry. Details about which information will be collected at each visit can be found in Table 1.

#### 2.5.1. Primary Outcome

The primary outcome is the proportion of patients having a reduction of 50% on global VAS, assessed with a 5-day pain diary between baseline (before lead implantation) and after each period of the crossover phase. Patients record their global, back and leg pain using a pain diary once a day, for a 5-day period within one week prior to each scheduled study visit.

#### 2.5.2. Secondary Outcomes

Secondary outcomes that will be compared between treatments and waveforms are changed between baseline and each follow-up visit in each of the following scores: pain intensity (VAS), pain surface (in cm^2^), percentage of pain covered with paresthesia and lead selectivity (percentage of paresthesia covering pain); paresthesia discomfort measured by an 11-point numeric rating scale (NRS) while sitting, standing and lying down; health-related quality of life measured by the EuroQol 5 Dimensions 5 Level (EQ-5D-5L) questionnaire; functional disability measured by the Oswestry Disability Index (ODI); anxiety and depression symptoms measured by the Hospital Anxiety and Depression Scale (HADS) and the Pain Catastrophizing Scale (PCS); and patient satisfaction measured by the Patient Global Impression of Change Scale (PGIC). Safety will be evaluated by adverse events (AE), serious adverse events (SAE) and device deficiencies from inclusion to 12-month follow-up.

Only for patients enrolled at Poitiers University Hospital: the effects of stimulation modalities on brain imaging will be measured by MRI Blood Oxygen Level-Dependent (BOLD) signal and Fractional Anisotropy (FA) resting state condition (stimulation switch off). Effects of stimulation on brain electrical activity will be measured by EEG signal of the posterior cingulate cortex (PCC), the pregenual anterior cingulate cortex (pgACC) and ventromedial prefrontal cortex (vmPFC), pgACC/vmPFC with stimulation switched on.

We will also evaluate stimulation efficacy using a published and clinically validated composite factor that allows for the multidimensional evaluation of patients [59]. Patients having at least three of the criteria listed below will be considered as good responders:

- Regaining functional capacity: having at least a 30% decrease in Oswestry Disability Index (ODI) percentage.
- Adequate global pain relief: having at least a 50% decrease in the Visual Analogic Scale (VAS).
- Improvement in quality of life: having at least a 0.2 increase in the EuroQol-5 Dimensions (EQ-5D) questionnaire.
- Decrease in psychological distress: having a decrease of at least 1.4 points in the Hospital Anxiety and Depression Scale (HADS) depression score.
- Decrease of at least 30% in pain surface (percentage of cm^2^): pain surface and its properties will be assessed using a pain mapping tool.
- Having a Patient Global Impression of Change (PGIC) score of at least 6/7.
- Drug intake: drug intake will be measured using the Medication Quantification Scale (MQS) and a reduction of at least 3.4 points will be considered as meaningful.

#### 2.5.3. Process Measures

At the screening and baseline visits, the following additional pieces of information are collected: general information, such as age, gender, date of birth, demographic and socioeconomic status; history of the disease, such as date of onset, date of management and etiology; medical and surgical history; analgesic treatments, concomitant medications and non-drug treatments; mapping of the thoraco-lumbar and lower limb painful areas; neuropathic pain assessment by clinician-administered diagnostic questionnaire (e.g., painDETECT Questionnaire (PD-Q)); and thoraco-lumbar MRI and brain imaging.

Patients who proceed to device implantation have the following information collected: mapping of the thoraco-lumbar and lower limb painful areas and paresthesia areas and recording of the various stimulation programs and stimulation parameters. The NeuroMapping tool is a validated quantitative tactile interface allowing the patient to delineate painful zones in the back and legs and to precisely map objective changes in pain coverage and SCS or DRGS performances [60,61].

Unscheduled patient visits could occur between scheduled study follow-up visits due to patient discontinuation from the study or device programming and management of any complication.

### 2.6. Procedures to Minimize Bias

To minimize selection bias, the randomization list will be prepared using the random selection program Ennov Clinical. Randomization numbers will be assigned in strict sequence; when a subject is confirmed as eligible for randomization, the next unassigned randomization number in sequence will be given. Randomization allocation will be concealed from the evaluator and subject, using a centralized automatic web-based data management system dedicated to the study and accessible to investigators by username and personal password (CS Online 7.5.10.11). Once assigned, the randomization assignment for the subject cannot be changed. Early departure from the study does not give rise to placement or reassignment of the rank of inclusion.

Due to the paresthesia generated by the TCS waveform, during the crossover period, patients will be under TCS waveform, whatever the type of stimulation. Consequently, patients will not be able to distinguish if they are under DRGS, SCS or DUAL stimulation. At the 3-month follow-up visit, all the patients will be switched to the burst waveform for a 1-month period while keeping the last allocated stimulation type in the randomized crossover arm. Patients will keep their stimulation in order to achieve optimal comparability between TCS and burst parameters.

To allow for investigator blinding, the evaluator will be different from the person who programs the IPG.

### 2.7. Statistical Analysis

#### 2.7.1. Sample Size and Power Calculations

In the ACCURATE study [24], the authors reported a significant difference between DRGS and conventional SCS for the treatment of the CRPS and causalgia using the following primary end point: “VAS decrease percentage ≥ 50% during lead trial and at 3-month follow-up and no incidence of neurological deficits due to stimulation”. They found that 81.2% of patients (56/69) responded to DRGS vs. 55.7% of patients (39/70) to SCS at 3-month follow-up. These data allowed us to estimate the necessary sample size that suffices to demonstrate a significant difference between the DRGS and conventional SCS. No data are available in the literature concerning DUAL stimulation. We estimate that DUAL stimulation superiority might arise from combining the effects of and mechanisms of action of both stimulation modalities.

With a level of significance at 0.05 and a power of 90%, we found that a number of patients set at 48 should be adequate to demonstrate a significant difference between the success proportions in SCS, DRG and DUAL stimulations in a six-arm crossover design.

Considering the possibility of a potential dropout (17%—lost to follow-up and possible explanted patients, which is the drop-out rate we observed in our previous studies [4,5]), and in order to have a sufficient number of patients allowing us to address secondary objectives, a total of 66 patients are planned to be included in the study.

#### 2.7.2. Main Statistical Analysis

Statistical analysis will be conducted using R software (Version 3.1.2, R Foundation for Statistical Computing, Vienna, Austria).

Data from phase A (12 patients) and phase B (54 patients) will be pooled (66 patients) for the main analysis.

Normality of distributions will be tested using the Shapiro–Wilk test. Either parametric or non-parametric methods will be used depending on the normality test results.

Our primary objective is to compare the efficacy of SCS, DRGS and DUAL stimulation at the crossover period based on the “50% VAS decrease” outcome.

Since our primary endpoint is a binary variable, we will use a mixed-effects logistic regression model, where the fixed effects are as follows: the treatment effect, the period effect and their interaction. The random effect is the random subject intercept. Fixed effects, their 95% confidence intervals and their significant level will be reported. Models will be estimated using restricted maximum likelihood estimation using the lmer function from the lmerTest package.

In case of significant effect of period and treatment, a two-step procedure will be conducted to assess pairwise differences between treatment effects. First, treatment effects will be estimated using a mixed-effects model as previously described, using data from each pair of treatments. Next, we will apply Holm–Bonferroni method to keep family-wise error under the 0.05 significance level.

In case the period effect is not significant, a Cochran Q test will be used to test the differences between the three groups. If the null hypothesis of the Cochran Q test is rejected, a McNemar test will be conducted for pairwise comparisons of the three groups. A Holm–Bonferroni correction will be applied to the *p*-values following the pairwise comparisons.

For the secondary outcomes, a two-factor repeated measure ANOVA (treatment + period factors) will be used to compare the continuous outcomes between the three treatments and a mixed effect logistic/multinomial regression model will be used to compare the categorical outcomes similarly to the analysis of the primary outcome. If the omnibus test’s *p*-value is significant, a pairwise comparison procedure with Holm–Bonferroni correction will be conducted.

At the 4-month period, the proportions of patients preferring the DRGS, the conventional SCS or the DUAL stimulation will be compared using a Chi-squared test.

Continuous outcomes at 4-month of burst DRGS, burst SCS and burst DUAL stimulations will be compared using an ANOVA or a Kruskal–Wallis test. In case the ANOVA (or Kruskal–Wallis test) is significant, a pairwise comparison will then be conducted using independent *t*-test or Mann–Whitney test depending on the variable distribution. A Holm–Bonferroni correction will be applied on the pairwise comparison.

The categorical outcomes will be compared using a Chi-squared test or an exact Fisher test when the Chi-squared assumptions are not met. A generalized mixed-effects model with a patient specific random intercept will be used to estimate the global treatment effect including the 6-month and 12-month follow-ups. The link function used in the model will depend on the type of the studied outcome (logistic or multinomial for categorical outcomes and no link function for continuous outcome).

The relationship between performance (percentage of pain covered with stimulation) and “induced” pain relief (global pain VAS) will be estimated by Kendall’s tau and its 95% confidence interval. The correlation between selectivity (percentage of paresthesia covering pain) and paresthesia discomfort will also be estimated using Kendall’s tau.

Mean energy consumption will be compared between the different types of stimulation using repeated measures ANOVA or a Friedman test.

Tonic stimulation will be compared to burst stimulation using patient last crossover stimulation modality. The comparison will be conducted using a paired *t*-test or a Wilcoxon signed-rank test using data from the last crossover period compared to the 4-month data using burst stimulation. VAS decrease will be compared between all the SCS modalities at 6-month and 12-month follow-ups using a two-factor ANOVA (preferred anatomical target x preferred stimulation type).

A sub-analysis of the sample will be conducted in order to assess the effect of burst waveform on the brain signals visualized using brain imaging data with and without burst stimulation.

Two interim analyses are planned. The first interim analysis will be conducted on data from the first 12 patients included (after the “Tonic” crossover period). In this first interim analysis, we will analyze only the safety criteria, the objective being to assess study feasibility. The second interim analysis will be conducted on data from the first 33 patients included (after the “Tonic” crossover period) and will be exclusively descriptive. No hypothesis testing will be conducted. This analysis will be conducted on primary and secondary outcomes. As the study is of long duration (3.5 years), we decided to include a second interim analysis to communicate the results to the scientific community by clearly indicating that this is an interim analysis and that the final analysis will be conducted on the total sample of 66 patients. This may lead to other projects and accelerate the progress of research on the stimulation of several anatomical targets. These results will have no impact on study completion. The final analysis on 66 patients will be considered for the report and the publications.

Only data from the crossover period will be used in the interim analysis. The statistical methods used in this interim analysis will be similar to the methods used in final analysis described above. No adjustment of the final analysis *p*-values will be conducted as the interim analysis results do not influence in any way the course or the conduct of the study.

A significance level of 0.05 will be considered in this study (after taking into account multiple testing corrections) and all tests will be two-sided.

All the analyses will be conducted based on an Intention-To-Treat (ITT) principle using complete cases. No missing data imputation will be conducted. An initial safety analysis will be conducted on the first 12 patients to assess the initial safety of implementing patients with both DRG and SCS stimulations. A final safety analysis will be conducted on the 66 patients. Frequencies of adverse events and deviations from protocol will be compared between the treatments following an ITT analysis.

#### 2.7.3. Extraction of Predictors of Long-Term Stimulation Efficacy

Long-term stimulation efficacy will be defined as being a “good responder”, according to the composite factor described in the Section 2.5.2 at 12-month follow-up.

The link between stimulation efficacy and each clinical, sociodemographic and psychological factor at baseline will be tested in a bivariate analysis. The link between quantitative variables and the long-term efficacy will be analyzed using *t*-tests or Wilcoxon test. The link between nominal qualitative variables and the long-term outcome will be tested using a Chi-squared test or an exact Fisher test depending on the number of patients per modalities. The link between ordinal variables and the long-term outcome will be tested using Cochran–Armitage test. Variables that are significant at a 5% level will be included in a multivariate logistic regression model where we will adjust for the type of stimulation and waveform by including interaction terms between the factors and the type of therapy.

## 3. Discussion

Based on a crossover design, our study is meant to provide evidence of SCS, DRGS or DUAL efficacy for treatment of chronic lower limb neuropathic pain and/or neuropathic back pain. The results will indicate comparative efficacy on pain characteristics, quality of life, functional disability, psychological distress and patient profile. In addition, mechanisms of action will be explored under “static conditions” (i.e., inactive stimulation/resting state) thanks to fMRI and under the influence of implanted stimulation (i.e., active stimulation condition) using live multisource-EEG analysis. Ultimately, our study aims to provide new insights and opportunities using burst waveforms applied to the DRG, marking a world first. Our sequencing will comprise three distinct phases, which should facilitate comparisons between stimulation spatial targets (SCS vs. DRGS vs. DUAL), comparisons between temporal waveforms (TCS vs. burst) and will ultimately respect patient preference regarding stimulation modalities, thereby documenting the variability of choices through incremental therapy personalization. Possible simultaneous synergy between SCS and DRG on the same patient might catalyze patient outcomes, in terms of neural structure targeting, pain relief, coverage, cortical perception and, ultimately, electrical consumption. That said, technological innovation using existing devices, which have not been intentionally designed for these challenges/this study, will underscore new technical limitations and reinforce the need for new device developments.

### 3.1. Intra-Subject Comparability

Within-subject comparisons will help to identify responder characteristics depending on stimulation target (SCS/DRG) and/or stimulation modality (TCS/burst).

From a technical standpoint, the lead trial procedure dedicated to identifying responders (whether the patient uses SCS and/or DRGS) will help us to avoid losing any potential patient, who could be identified as a non-responder with one of the two techniques. Additionally, the choice to implant the leads under awake anesthesia will facilitate optimal paresthesia coverage, since the initial phase will optimize sweet-spot coverage thanks to patient intra-operative feedback provided by objective tactile interface and mapping data [60,61]. It bears mentioning that the choice to use this “optimized” placement for SCS and DRG leads, to apply stimulation, is deliberate, and that what might correspond to optimal tonic stimulation paradigm might not correspond to optimal burst stimulation settings. Our study design is not intended to address this question. While similar procedures have been observed in previous RCT designs, which have assessed different stimulation modalities [31,62], this question needs to be examined in specific future research.

The A/B/A + B comparative design will compare two different spatial neurostimulation targets, using the same technology, delivered to the same patient, in a double-blind manner, which is quite rare in neurostimulation studies [63]. However, our study design will not be applicable when comparing the two different temporal modalities (see below/study limitations).

### 3.2. “High-Fidelity” Composite Multidimensional Patient Assessment

Our study is also intended to build a composite score based on NRS, pain mapping, EQ-5D, ODI, HADS and PCS. This score will provide a comprehensive and global picture of patient health evolution through the follow-up period, as previously reported in recent works [59,64]. In an attempt to reflect the impact of chronic pain not only on the patient’s nervous system, but also on patient function, cognition, social life, etc., as has been the substrate of the bio-psycho-social concept and pain matrix theory, and the composite score will take into account not only pain intensity, but also many other pain dimensions such as psychological distress, functional disability and influence on quality of life [59]. In addition to being simple pieces of the complex puzzle of pain, these factors have been shown to be crucial, the objective being to determine with accuracy the profile of a patient for whom health quality of life can be influenced primarily by pain intensity and functional disability, or by psychological distress [65].

Some authors would insist on the subjectivity, and consequently the lack of reliability, of patient’s reported outcomes in comparison between two techniques or two modalities, especially as the double-blind design remains difficult to maintain in a study in which one strategy can be perceived by the patient and the other not. This could euphemize the added value and even the rationale of composite indexes. In our opinion, a need for objectivity fully justifies combining patient clinical data with a functional imaging approach and live exploration of the neural networks involved during stimulation.

Identification of cortical mechanisms of action would also provide substantial knowledge about SCS- and DRGS-related induced cerebral plasticity. Recently, MRI studies have suggested that decreased brain activity in the medial thalamus, the insula, the somatosensory cortex and the anterior cingulate cortex after SCS is associated with good outcomes [66,67,68,69,70,71]. Nagamachi et al. [66] reported that poor SCS responders showed thalamic activation increase, whereas good SCS responders showed no activation in the thalamus. While these studies provide crucial information about SCS implication at the cortical level, MRI did not assess patients under active SCS. To bridge this gap, a high-resolution multi-channel (128 channels) MRI-compatible EEG device will be used in our study. In their source-localized EEG study, de Ridder and Vanneste highlighted different cortico-spinal mechanisms involved in TCS and burst modalities [48]. The authors reported that TCS would mainly modulate the ascending lateral pathway (coding for pain perception) and descending inhibitory pain pathway, while burst would additionally modulate the medial pain pathway (coding for attention to pain, emotion and motivation). Although this pilot study brought new fundamentals to better understand the mechanisms involved in SCS, methodological considerations reveal some limitations. First, this study was performed in only five patients. This clearly nuances and euphemizes the conclusion of the authors. The authors used a 19-EEG channel system, which nowadays can be considered as suboptimal to determine localization source detection accurately [72,73]. Moreover, EEG measurements were not combined with fMRI study, which could enhance result relevance and understanding. This reinforced our choice to use an MRI compatible EEG device.

### 3.3. A Focus on DRG. Indications and Technical Considerations

While most SCS indications are dedicated to back and leg pain neuropathic components in refractory PSPS-T2 (FBSS) patients worldwide [2,4,5,6,8], DRGS has been validated on more focal pain, CRPS and causalgia [22,23,24]. Therefore, it appears interesting to put DRGS into perspective with main SCS indication, applying a new spatial target (DRG) to the main population targeted for neurostimulation (SCS), for several reasons. First, it will be of interest to determine whether DRGS will be more specific than SCS in terms of radicular selectivity, since the main source of the problem often arises from selective nerve root lesions (PSPS-T2). Second, we wish to know whether DRGS, on the contrary, has the same potential as SCS to generate significant coverage of the painful axial component, for which innervation depends on the posterior branches of the affected nerve roots and for which pain typology is characterized by a constant mix of neuropathic and mechanical features. Functional imaging and MOA explorations will be precious in this context.

In parallel, while we may suppose that DRGS could become a good indication for PSPS-T2 after clinical validation, several technical considerations need to be discussed. Indeed, a significant proportion of PSPS-T2 patients, who have undergone several (and sometimes major) spine surgeries, develop epidural fibrosis at the foraminal level, corresponding to the potential targeted DRG area to be stimulated. Posterior instrumentation at adjacent levels can become serious technical obstacles to DRG trans-grade approach and represent an impossible Everest to climb, even for experienced implanters. To overcome these potential barriers, we will perform ultra-selective transforaminal anesthetic blocks using contrast agents before implantation to determine whether the foramen is accessible for an implant and to confirm that the nerve root is free of entrapment. The relevance of such a procedure, as recommended or as a mandatory DRGS pre-implantation test, will be discussed in light of our results. This might also help to develop an extra-foraminal needle approach and/or a surgical minimally invasive technique to target DRG “from outside to inside of the spinal canal” for some difficult cases. The choice to consider DRG implantation in PSPS-T2 patients in this study should bring valuable information.

### 3.4. A Focus on Burst, as a Combined Waveform to DRGS, but Also to SCS+DRGS, as a New Hybrid Stimulation Modality

Applying burst at the DRG site appears of major interest and has not been published yet, alone or in combination with burst SCS. Many speculations will legitimately arise from this synergistic concept, with an emphasis on electrical consumption. Even though DRGS requires usually very low energy (due to the proximity of the neural target), the combination with burst SCS could override IPG capabilities. Moreover, the existing sources have not been developed to generate DRG burst stimulation. While DRG remains a structure that is highly sensitive to aggressive stimulation, in case of overstimulation, it would respond immediately, with a painful sensation for the patient, which might not be compatible with permanent burst applied to DRG. This potential concern would appear even more striking insofar as it remains strictly impossible to generate two different synchronous burst programs on two different leads (at two different sites in this study) at the moment using existing devices. A final interest of this study will be to clarify, depending on the results, the need, or not, to develop a new generation of IPGs to bridge these gaps.

### 3.5. Study Limitations

Despite potential interest, this study has several limitations. First, as mentioned above, the comparison between TCS (involving a paresthetic effect) versus burst (a paresthesia-free modality) will not be performed under strict and homogeneous blinded conditions, by sequentially testing the different stimulation modalities. However, burst will be applied for the three groups (SCS, DGRS and DUAL) between the 3rd and the 4th month after randomization, which will allow for the comparison of paresthesia-free conditions as a second step, in a second phase. This should not interfere with our main endpoint, at 3-month follow-up, involving TCS and paresthesia for each group of stimulation, in a strict double-blind manner. Second, this crossover design will not allow us to determine long-term efficacy of each stimulation modality, since multiplying the number of modalities to evaluate implies proportionally decreasing the length of the follow-up period for financial and ethical reasons. An alternative point of view would counterbalance this loss of potential valuable information with the fact that this design can also be considered as “added value” for the patient, who will be able to choose his preferred spatial target and waveform, thereby enhancing treatment adherence. Following this perspective, we will collect maximal follow-up for the stimulation modality, which will reflect the patient’s preference. Third, the absence of wash-out period will not allow us to claim that one modality could not induce a carryover effect. However, the 1-month period used for each modality should cancel out this potential carryover effect. Fourth, mechanisms of actions will be assessed at the supraspinal level, even though some modulations can occur at the spinal level. In their in vitro simulation study, Zannou et al. [74,75] interestingly reported tissue heating when stimulation was applied at a high-frequency modality, which that may impact more or less long-term clinical outcomes. Realistic anatomical computational models of SCS are crucial tools and provide opportunities to better understand the role of complex tissue compartments in shunting the current applied to the potential neural targets [74,75,76].

## 4. Conclusions

The BOOST DRG study seeks to provide clinical comparative evidence of SCS, DRGS or SCS+DRGS combination efficacy, using either tonic conventional or burst stimulation, in patients presenting with refractory chronic lower limb neuropathic pain or/and neuropathic back pain. Patient assessment will be based on multiple criteria including pain intensity, pain mapping, quality of life, functional disability, psychological distress, catastrophizing and differential cerebral mechanisms of action explored with functional brain imaging coupled with 128-channel MRI-compatible EEG.

Our ambition is to capture the potential added value of one novel SCS temporal modality, burst, potentially combined with new spatial targets focusing on DRG, and to bring new insight for patient pain assessment in RCTs, by using multi-dimensional composite objective measurement tools, as a new “high-fidelity” standard for pain studies.

## Figures and Tables

**Table 1:** Radio-clinical assessment calendar.

	Inclusion Visit	Implantation Visit (Within 2 Months after Inclusion)		Visit M1 [1 Month ± 1 Week /M0]	Visit M2 [1 Month ± 1 Week /M1]	Visit M3 [1 Month ± 1 Week /M2]	Visit M4 [1 Month ± 1 Week /M3]	Visit M6 [2 Months ± 2 Weeks /M4]	VisitM12 [6 Months ± 3 Weeks /M6]
		Lead Implantation Visit	Visit M0 (IPG Implantation + Randomization)						
Patient information and consent form (R)	✓								
Inclusion and non-inclusion criteria (R)	✓								
Exclusion criteria (R)	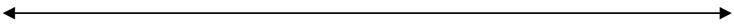								
Socio-demographic data (C)	✓								
Medical and surgical history (C)	✓								
Spine MRI + thoraco-lumbar X-rays (front + profile) ^1^ (C)	✓								
painDETECT Questionnaire (C)	✓								
Brain imaging (fMRI/EEG) ^2^ (R)	✓ ^3^					✓	✓		
VAS (C) *	✓								
ODI(C) *, HADS(C) *, PCS(R) *, EQ5D-5L(C) *	✓			✓	✓	✓	✓	✓	✓
Spine X-ray (C)		✓(post-impl)							
Mapping of the painful territory (C) *	✓	✓	✓(post-impl)	✓	✓	✓	✓	✓	✓
Mapping of paresthesia coverage (C) *		✓(post-impl)	✓(post-impl)	✓	✓	✓	✓	✓	✓
Stimulation parameters and programming (C)		✓(post-impl)	✓(post-impl)	✓	✓	✓	✓	✓	✓
Patient satisfaction (PGIC) (C) *			✓(post-impl)	✓	✓	✓	✓	✓	✓
Paresthesia discomfort 11-point NRS (C) *				✓	✓	✓	✓	✓	✓
5-day VAS diary (R)	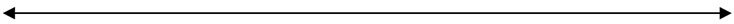								
Concomitant medications ^3^ (C)	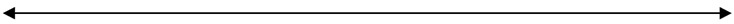								
AE/SAE/Device deficiencies	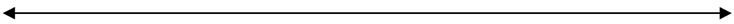								

^1^ Only if not already available, dating >2 years or since the last spinal surgery; ^2^ Only for patients from Poitiers University Hospital; ^3^ To schedule between inclusion and lead implantation visit; * to perform through a touchscreen tablet; (R): specific to the research; (C): as per standard care. The arrow indicates exclusion criteria was performed in every visit.

## Data Availability

Not applicable.
